# Hepatitis C virus prevalence and genetic diversity among pregnant women in Gabon, central Africa

**DOI:** 10.1186/1471-2334-8-82

**Published:** 2008-06-17

**Authors:** Guy-Roger Ndong-Atome, Maria Makuwa, Richard Njouom, Michel Branger, Francoise Brun-Vézinet, Antoine Mahé, Dominique Rousset, Mirdad Kazanji

**Affiliations:** 1 Laboratoire de Virologie, Centre International de Recherches Médicales (CIRMF), BP 769, Franceville, Gabon; 2 Laboratoire de Virologie, Centre Pasteur du Cameroun, Yaounde, Cameroon; 3 Service de Virologie, Centre Hospitalier Bichat-Claude Bernard, Paris, France; 4 Programme National de Lutte contre le Sida, Libreville, Gabon; 5 Service de Coopération et d'Action Culturelle, French Embassy, BP 2105, Libreville, Gabon; 6 Réseau International des Instituts Pasteur, Institut Pasteur, 28 rue du Dr Roux, 75015 Paris, France

## Abstract

**Background:**

Hepatitis C virus (HCV) infection is a major global public health problem in both developed and developing countries. The prevalence and genetic diversity of HCV in pregnant women in Gabon, central Africa, is not known. We therefore evaluated the prevalence and the circulating genotypes of HCV in a large population cohort of pregnant women.

**Methods:**

Blood samples (947) were collected from pregnant women in the five main cities of the country. The prevalence was evaluated by two ELISA tests, and the circulating genotypes were characterized by sequencing and phylogenetic analysis.

**Results:**

Twenty pregnant women (2.1%) were infected with HCV. The seroprevalence differed significantly by region (p = 0.004) and increased significantly with age (p = 0.05), being 1.3% at 14–20 years, 1.1% at 21–25 years, 1.9% at 26–30 years, 4.1% at 31–35 years and 6.0% at > 35 years. Sequencing in the 5'-UTR and NS5B regions showed that the circulating strains belonged to genotypes 4 (4e and 4c).

**Conclusion:**

We found that the HCV seroprevalence in pregnant women in Gabon is almost as high as that in other African countries and increases with age. Furthermore, only genotype 4 (4e and 4c) was found. More extensive studies aiming to evaluate the prevalence and heterogeneity of HCV genotypes circulating in the general population of the country are needed.

## Background

Hepatitis C virus (HCV) infection is a major global public health problem in both developed and developing countries. More than 170 million people are chronically infected with HCV worldwide, and the infection results in the development of chronic liver diseases, including liver cirrhosis and hepatocellular carcinoma. HCV has been clustered into six distinct genotypes and numerous subtypes on the basis of nucleic acid sequence [1]. Genotyping of HCV strains is an important tool for epidemiological and clinical purposes; in particular, examination of sequence diversity can aid understanding of different patterns of serological reactivity, virulence and response to treatment [2,3].

Genotypes 1, 2 and 3 are distributed widely throughout western countries, whereas types 5 and 6 are largely confined to South Africa and southeast Asia, respectively, and type 4 was first found predominantly in the Middle East [4] and then in central Africa [5-7]. Data on HCV prevalence and molecular epidemiology are available for only a few African countries. In Gabon, central Africa, the seroprevalence was previously reported to be up to 6.5% in the general population of the eastern part of the country [8], and genotype 4 was found by screening a few sequences [7]. These data are based on small groups of individuals and third-generation enzyme-linked immunosorbent assays (ELISAs), molecular (reverse transcriptase polymerase chain reaction (RT-PCR)) assays having rarely been used. Furthermore, no data are available on the presence of circulating HCV or the genotypes in pregnant women in Gabon. A serological and molecular study was therefore undertaken on a large population cohort of pregnant women in the five main cities of the country.

## Methods

### Area and study population

Gabon occupies 270 000 km^2 ^in the Gulf of Guinea near the equator, with tropical forest covering three-quarters of the territory. The population was estimated in 2003 to be 1 273 000 persons, belonging to more than 40 ethnic groups.

**Figure 1:**
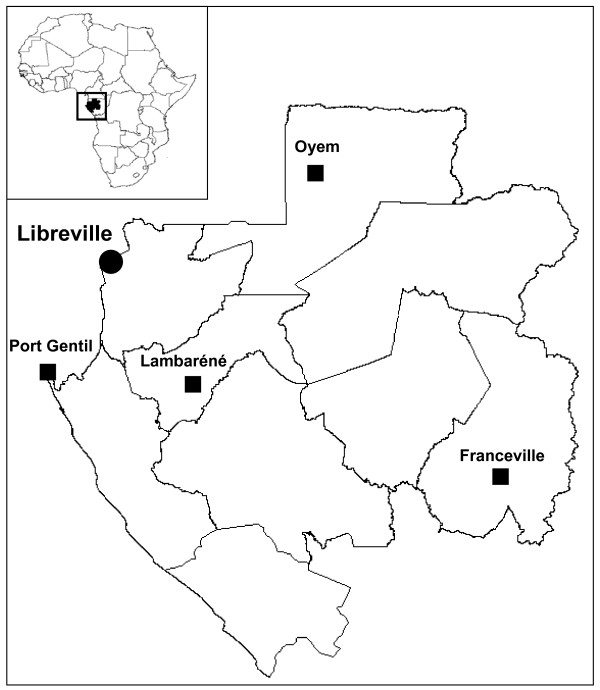
Map of Gabon in central Africa with the selected sentinel sites at which blood was collected from pregnant women.

To determine the prevalence and the circulating genotypes of HCV in pregnant women in Gabon, five sentinel sites were selected: Libreville, the capital, in the north-west; Port-Gentil, the port and economic capital in the west; Lambaréné, in the centre of the country; Franceville in the south-east; and Oyem in the north-east (Figure 1). Between January and March 2005, blood samples were collected from all pregnant women at their first antenatal examination, after full informed consent had been obtained. The samples were anonymous, but age and geographic origin were retained. The study obtained ethical clearance from the public health authorities (Ministry of Public Health and the "Programme National de Lutte contre le Sida" in Gabon).

### Serological tests

All samples were screened for anti-HCV antibodies with a commercial third-generation ELISA (Monolisa, anti-HCV Plus version 2, Bio-Rad, Marne-La-Coquette, France). The results of the assay were expressed quantitatively as the ratio of the optical density of the test sample to the calculated cut-off absorbance, as recommended by the manufacturer. Sera with ratios > 1.1 were considered to be positive, sera with R values of 0.9–1.1 were recorded as indeterminate, whereas those with R values < 0.9 were considered negative. Positive and indeterminate sera were re-tested with another third-generation ELISA assay, Innotest HCV Ab IV (Innogenetics NV HCV, Gand, Belgium). Samples were considered HCV positive when they were positive in the two ELISA tests.

### HCV amplification and molecular and phylogenetic analysis

Viral RNA was extracted from 140 μl of plasma with a Qiamp^® ^viral mini kit according to the manufacturer's protocol (Qiagen, Courtaboeuf, France). The extracted RNA was used as a template and amplified by nested RT-PCR with primers specific to the 5'UTR (5' non-coding region), as described previously [9,10]. Reverse transcription and first amplifications were performed with a Titan One Tube RT-PCR kit (Roche Diagnostics, Mannheim, Germany), according to the manufacturer's protocol. A portion of the NS5B region was also amplified with degenerated primers, as described previously [11], and then sequenced according to standard procedures. The HCV sequences and phylogenetic results were studied as reported previously [11]. The GenBank accession numbers of the new NS5b sequences are EU0334530 to EU0334534, and those of the 5'UTR sequences are  to EU0334545.

### Statistical analysis

Statistical analysis was performed by the chi-squared test with Yates' correction, and the prevalences and odds ratios were calculated. The corresponding 95% confidence intervals were reported as measures of statistical significance. Analyses were performed with Epi-Info (version 6.04dfr, ENSP-Epiconcept-InUS, 2001).

## Results

### HCV prevalence in pregnant women

Between January and March 2005, sera from all 947 pregnant women recruited at the five sentinel sites were screened for HCV antibodies with the two ELISA tests; 20 pregnant women (2.1%) were found to be seropositives for HCV (Table 1). The seroprevalence differed significantly by region (p = 0.004), with the highest prevalence (4.9%) in Franceville (Haut Ogooué region in the south-east) and no cases in Port Gentil. There was a significant increase in HCV seroprevalence with age (p = 0.05), being 1.3% at 14–20 years, 1.1% at 21–25 years, 1.9% at 26–30 years, 4.1% at 31–35 years and 6.0% at > 35 years (Table 1).

**Table 1:** Prevalence of antibodies to hepatitis C virus (HCV) in pregnant women in Gabon (central Africa) by geographical area and age group

Variable	HCV antibodies			
	
	No. positive/no. tested	%	Odds ratio	95% CI
Sentinel site				
Port Gentil	0/168	0		
Lambaréné	3/331	0.9	0.33	0.1–1.13
Libreville	5/199	2.5	1.29	0.46–3.59
Oyem	3/68	4.4	2.39	0.68–8.37
Franceville	9/181	4.9	3.64	1.49–8.92
				
Age range (years)				
14–20	4/293	1.3	0.57	0.19–1.72
21–25	3/266	1.1	0.46	0.13–1.58
26–30	4/202	1.9	0.94	0.31–2.84
31–35	5/120	4.1	2.4	0.86–6.73
> 35	4/66	6.0	3.5	1.15–10.94
				
All	20/947	2.1		

### HCV RNA detection and molecular analysis of circulating genotypes

HCV RNA targeting 5'-UTR was found in 13 of the 20 positive samples, showing that 1.3% of the pregnant women were viraemic. Sequencing in the 5'-UTR region was successful for 11 RNA-positive isolates (Table 2). BLAST searches of the 5'-UTR sequences classified the 11 strains as genotype 4. Of these, nine belonged to genotype 4e, with 99–100% homology with the reference sequences GAB809 (L29625) and CAM73 (L29591) from Gabon and Cameroon, respectively [12]. The other two sequences belonged to genotype 4c, with 98% homology with the reference sequences Z6 (M84862) and GB358 (L29608) from Zaire and Gabon, respectively [13] (Table 2).

**Table 2:** Epidemiological status and results of molecular screening and 5'UTR sequence analysis for the 20 serologically HCV-positive pregnant women in Gabon, central Africa.

Sample	Age (years)	Region	PCR 5'UTR	BLAST % homology with accession number		Subtype	Accession number
Gab104	32	Lambaréné	Positive	98	L29608	4c	EU334535
Gab121	18	Lambaréné	Negative	-	-	-	
Gab977	26	Lambaréné	Positive	99	L29625	4e	EU334536
Gab652	16	Libreville	Positive	100	L29625	4e	EU334537
Gab704	28	Libreville	Positive	99	L29625	4e	EU334538
Gab705	21	Libreville	Negative	-	-	-	
Gab757	21	Libreville	Negative	-	-	-	
Gab770	33	Libreville	Positive	100	L29591	4e	EU334539
Gab1089	23	Franceville	Positive	100	L29625	4e	EU334540
Gab1115	40	Franceville	Negative	-	-	-	
Gab1139	26	Franceville	Positive	99	L29625	4e	EU334541
Gab1148	19	Franceville	Positive	98	M84862	4c	EU334542
Gab1174	29	Franceville	Positive	100	L29591	4e	EU334543
Gab1192	30	Franceville	Negative	-	-	-	
Gab1213	34	Franceville	Negative	-	-	-	
Gab1228	15	Franceville	Positive	99	L29625	4e	EU334544
Gab1229	31	Franceville	Positive	NA	NA	NA	
Gab1664	25	Oyem	Negative	-	-	-	
Gab1669	19	Oyem	Positive	NA	NA	NA	
Gab1670	19	Oyem	Positive	99	L29625	4e	EU334545

NA, not available; only partial sequence of the amplicon could be obtained.

**Figure 2:**
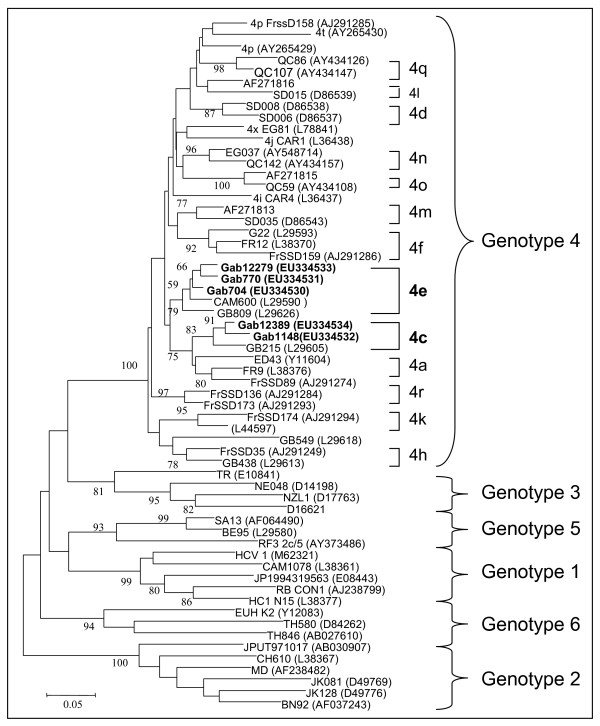
**Phylogenetic analysis of HCV NS5B sequences**. A neighbour-joining phylogenetic tree was constructed from Gabonese isolates from pregnant women, and published reference sequences for the various HCV genotypes were obtained from GenBank. The Kimura two-parameter method of estimating genetic distance was used. Numbers next to the nodes of the tree represent bootstrap values (1000 replicates). In genotype 4, sequences found in Gabon are indicated in bold. The GenBank accession numbers of the new NS5b sequences of HCV from Gabon are EU0334530 to EU0334534.

To confirm the circulating genotypes obtained with 5'-UTR sequences and for phylogenetic analysis, the NS5B regions of three strains were amplified and sequenced. We also amplified and sequenced two new positive samples obtained from routinely screened pregnant women in the region with the highest HCV prevalence (Franceville). These two samples were also included in the phylogenetic analysis. Sequence analysis of the NS5B region confirmed that the strains from pregnant women were closely related to genotypes 4e and 4c, with 94.5% and 93.7% homology with other strains from Gabon, as described previously [12]. Phylogenetic analysis of the NS5B region confirmed the sequence homology analysis, showing that the strains from pregnant women in Gabon belonged to genotypes 4c and 4e (Figure 2). Within genotype 4e, our strains clustered with strains from Cameroon and with other strains from Gabon described previously [12]. In genotype 4c, the strains from Gabon were closely related to other strains identified previously by Stuyver et al. (1994).

## Discussion

We found an overall seroprevalence of HCV in pregnant women in Gabon of 2.1%, which is similar to that in other African countries, such as Cameroon (1.9%) [14] and Burkina Faso (2%) [15]. The prevalence in our study was lower than that described previously in the general population of eastern Gabon (6.5%) [8]. This difference could be related to age, as we observed an increasing prevalence with age and low prevalences in the youngest age groups (Table 1). Similar observations have been made in previous studies in several African countries, including Cameroon and the Democratic Republic of the Congo, in both pregnant women and the general population [14,16]. Although studies to evaluate the routes of HCV transmission in Africa are needed [17], ritual procedures, such as scarification and tattooing, which are common tribal customs in various areas of Gabon, may facilitate transmission [18]. Furthermore, in rural health centres in Gabon, drugs such as antibiotics are commonly given by intramuscular injection, and the sterilization procedures are often inadequate, increasing the risk for HCV transmission. People are therefore exposed to new infection throughout their lives. This might explain in part the increasing prevalence of HCV with age.

We also showed that HCV seroprevalence differed significantly by region. Many factors can influence the transmission of HCV in Africa, including environmental, ethnic, ritual (see above), economic and genetic factors. In Egypt, it was reported that transmission of HCV was related to extensive antischistosomiasis injection campaigns [19]. It is not known whether previous vaccination campaigns in Gabon had the same effects in various areas of the country. Further epidemiological, molecular and genetic studies are required to determine the factors responsible for the high prevalence of HCV in some regions of Gabon.

Of the 20 seropositive women, seven were ELISA-positive but RNA-negative. These seven samples also had a low ratio in the two ELISA tests. It has been reported previously that during the natural history of HCV infection, 20–30% of infected individuals eliminate the virus spontaneously [20,21]. Results similar to ours were obtained in studies conducted in Cameroon and Japan [14,22]. The presence of HCV antibodies in the pregnant women in our study might have been due to a previous infection.

We also showed that the HCV circulating in pregnant women in Gabon belongs to genotype 4 (4e and 4c). In a previous study, Xu et al. [7] published three sequences of HCV selected from 1172 persons in the general population of Gabon. Sequence comparisons showed that these three sequences were closely related to genotype 4, and the authors suggested that they might be related to genotype 4c or to 4d as described by Buck et al. [13]. In our study, the sequences of 11 samples were also found to be closely related to genotypes 4c and 4e. These two studies thus confirm that genotype 4 is the only genotype circulating in Gabon.

In our study, we also found that 4e predominates in five widely separated geographical regions of the country. Sub-Saharan Africa is known to have a wide diversity of genotypes 1, 2, and 4 and a remarkable diversity of subtypes [6,11,23]; genotypes 4 and 5 predominate in several African countries [24-26], and genotype 4f has been described in Cameroon. We found only genotypes 4e and 4c in pregnant women in Gabon. Indeed, the plasma samples used in the present study, obtained after screening for HIV, HTLV, HBV/HDV and HEV, were insufficient to perform more sequence analyses. However, the two strains obtained from further routinely screened pregnant women in the region of the highest HCV prevalence (Franceville) confirm, however, that the circulating strains in this population belong to genotype 4 (4c and 4e). A larger study in the general population is needed to better characterize the circulating genotypes.

## Conclusion

Our study represents the first serological and molecular epidemiological investigation on HCV in a homogeneous population in Gabon. We showed that the HCV seroprevalence in pregnant women in Gabon is almost as high as that in other African countries and increases with age. Thus, preventive measures to decrease the spread and transmission of HCV in Gabon are warranted. These measures should include systematic HCV screening of pregnant women, in order to counsel them about the risk for HCV transmission and the development of associated diseases.

Only genotype 4 (4e and 4c) was found in these pregnant women. More extensive studies aiming to evaluate the heterogeneity of HCV genotypes in samples collected from the general population of the country are needed.

## Competing interests

The authors declare that they have no competing interests.

## Authors' contributions

G–RN-A, MM and MB carried out the serological and molecular studies. RN and DR participated in sequence alignments and phylogenetic analysis. AM collected the sera and compiled the epidemiological data. FB–V and MK participated in the design of the study, the statistical analysis and the drafting of the manuscript. All authors read and approved the final version of the manuscript.

## Pre-publication history

The pre-publication history for this paper can be accessed here:


